# Optimizing target inactivation to treat multidrug-resistant *Escherichia coli* with NDM and PBP3 mutations: “going the extra mile”

**DOI:** 10.1128/aac.00887-25

**Published:** 2026-02-20

**Authors:** Claudia Fabrizio, Felice Valzano, Simone Giuliano, Elisabetta Morelli, Daniela Serio, Giovanni Battista Buccoliero, Maurizio Cervellera, Gianfranco La Bella, Fabio Arena, Andrea M. Hujer, Magdalena A. Taracila, Steven H. Marshall, Robert A. Bonomo, Carlo Tascini

**Affiliations:** 1 Infectious and Tropical Diseases Clinic, San Giuseppe Moscati Hospital 220578 , Taranto, Italy; 2 Department of Clinical and Experimental Medicine, University of Foggia 18972, Foggia, Italy; 3 Infectious Diseases Clinic, Department of Medicine (DMED), University of Udine 9316, Udine, Italy; 4 Clinical Pathology Unit, SS Annunziata Hospital 73127 , Taranto, Italy; 5 Intensive Care Unit, SS Annunziata Hospital 73127 , Taranto, Italy; 6 General Surgery Unit, SS Annunziata Hospital 73127 , Taranto, Italy; 7 Istituto Zooprofilattico Sperimentale della Puglia e della Basilicata 92710, Foggia, Italy; 8 Microbiology and Virology Unit, Ospedali Riuniti 18567, Foggia, Italy; 9 Department of Medicine, Case Western Reserve University School of Medicine 12304, Cleveland, Ohio, USA; 10 Research Service, Louis Stokes Cleveland Department of Veterans Affairs Medical Center 20083, Cleveland, Ohio, USA; 11 Department of Pharmacology, Case Western Reserve University School of Medicine, Cleveland, Ohio, USA; 12 Department of Molecular Biology and Microbiology, Case Western Reserve University School of Medicine, Cleveland, Ohio, USA; 13 Department of Biochemistry, Case Western Reserve University School of Medicin, Cleveland, Ohio, USA; 14 Department of Proteomics and Bioinformatics, Case Western Reserve University School of Medicine, Cleveland, Ohio, USA; 15 CWRU-Cleveland VAMC Center for Antimicrobial Resistance and Epidemiology (Case VA CARES), Cleveland, Ohio, USA; Houston Methodist Hospital and Weill Cornell Medical College, Houston, Texas, USA

**Keywords:** double β-lactam combinations, aztreonam, imipenem/relebactam, CMY, penicillin binding protein3, NDM-5, *E. coli*

## Abstract

A 65-year-old man without identifiable risk factors for multidrug-resistant pathogens was admitted with peritonitis, isolating NDM-producing *Escherichia coli* from a rectal swab and intraoperative samples. After surgery, ceftazidime-avibactam/aztreonam was administered. Due to poor clinical response, he was switched to imipenem-relebactam/aztreonam, resulting in a successful outcome. Whole-genome sequencing detected *bla*_NDM-5_ and *bla*_CMY-148_ β-lactamases, PBP3 YRIN insertion, and mutated *cirA* gene. This case illustrates the importance of considering different mechanisms of resistance when choosing combination therapy.

## INTRODUCTION

Infections caused by multidrug-resistant (MDR) organisms, including NDM-producing *Escherichia coli*, represent a challenging clinical scenario, despite the availability of novel therapies. These strains are emerging in community-acquired infections even in the absence of risk factors for the acquisition of MDR pathogens, especially in endemic countries (1). Unfortunately, such strains are now being described worldwide (2, 3). Some isolates also display penicillin-binding protein 3 (PBP3) alterations, impairing the activity of cephalosporins and aztreonam (ATM) (4) and leaving few treatment options. Herein, we report a case of complicated community-acquired intra-abdominal infection (cIAI) treated with imipenem/relebactam and aztreonam caused by NDM-5-producing *E. coli* harboring an uncharacterized *bla*_CMY-148_, a *cirA* mutation conferring resistance to cefiderocol, and a tetrapeptide (YRIN) PBP3 insertion.

## CASE PRESENTATION

A 65-year-old man was admitted to Taranto Hospital (Italy) with peritonitis due to ruptured diverticulum. His medical history included mild pulmonary fibrosis (not requiring treatment) and the most recent hospitalization (elective lung nodulectomy) dated back to 4 years before the current admission. The last invasive procedure was a colonoscopy (2 years before hospitalization). He never traveled outside Europe and did not have a known history of isolation of drug-resistant pathogens. Previous antibiotic exposure only included short courses of orally administered antibiotics within 3 months before the current episode, for the treatment of mild respiratory infections; in particular, he received a short course of amoxicillin-clavulanate and, after 4 weeks, another short course of levofloxacin (no longer than 4–5 days per regimen).

**Fig 1:**
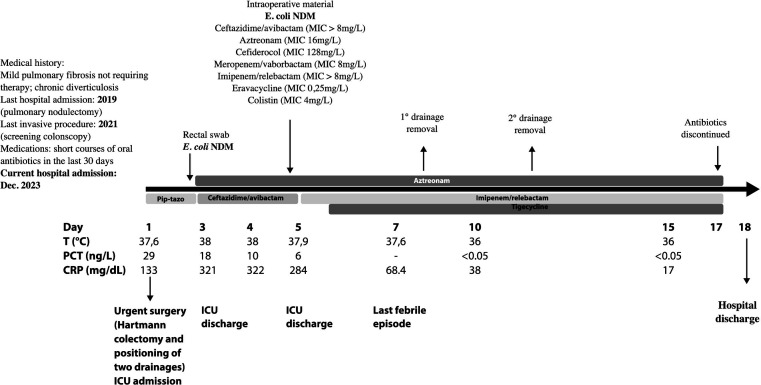
Clinical course of the *E. coli* infection. T: temperature; PCT: procalcitonin; CRP: C-reactive protein; Pip-tazo: piperacillin-tazobactam; ICU: intensive care unit; NDM: New Delhi metallo-β-lactamase.

Twenty-four hours post-admission, the patient underwent Hartmann hemicolectomy, placement of two drains, and piperacillin-tazobactam 4.5 g q6h was started empirically. Subsequently, he was admitted to the intensive care unit (ICU) (Fig. 1). On day 2 post-hospital admission, a rectal swab for a carbapenemase-producing microorganism (collected as part of routine ICU screening) yielded NDM-producing *E. coli* (cultured on selective medium, followed by characterization with molecular method: Xpert Carba-R, Cepheid, Sunnyvale, CA). As a result, ceftazidime-avibactam (CZA) 2.5 g q8h plus ATM 2 g q8h was initiated. An NDM-producing *E. coli* isolate was at4rlso recovered from intraoperative sample (peritoneal fluid), whereas blood cultures were negative.

**Fig 2:**
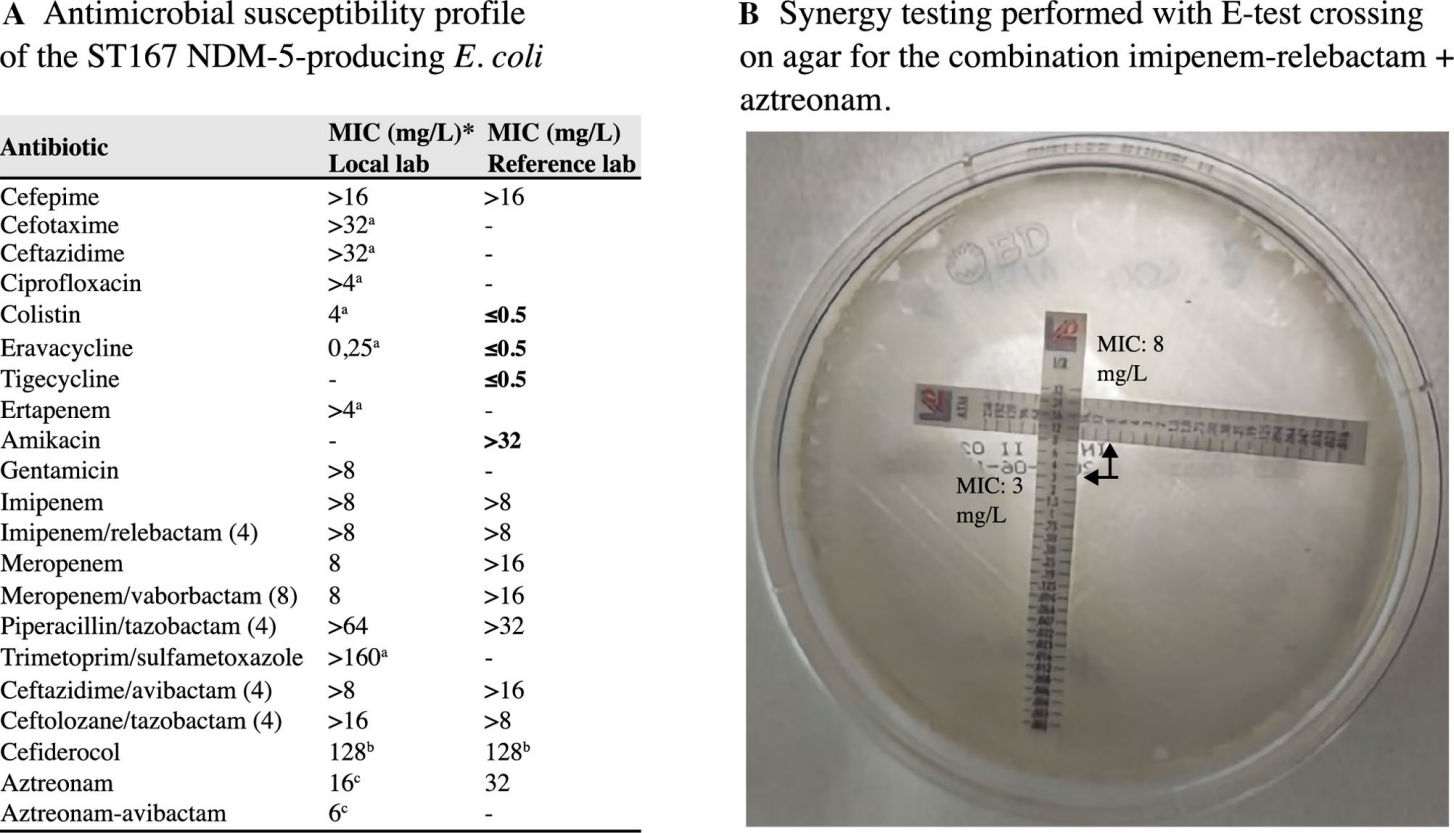
(**A**) Antimicrobial susceptibility profile of the ST167 NDM-5-producing *E. coli*. (**B**) Synergy testing performed with E-test crossing on agar for the combination I/R + ATM. *Unless otherwise specified, molecules were tested with automated systems (Vitek 2, Biomerieux), then verified and confirmed by broth microdilution (EUMDROX panel, Thermofisher). ^a^Molecule tested with automated system only. ^b^Cefiderocol susceptibility was tested by BMD method in iron-depleted cation-adjusted Mueller–Hinton broth. Results were interpreted according to the most recent EUCAST clinical breakpoints (EUCAST breakpoints, v. 14.1, 2024). ^c^Aztreonam MIC was also confirmed by eTest with MIC strip; ATM–avibactam was tested with eTest. For this combination, there is currently no breakpoint available, although a tentative breakpoint has been recently proposed by Sader et al. (5).

According to the AST results, the isolate exhibited resistance to all β-lactams, including novel β-lactam/ β-lactamase inhibitor (BL/BLI) combinations (according to EUCAST criteria: https://www.eucast.org/fileadmin/src/media/PDFs/EUCAST_files/Breakpoint_tables/v_15.0_Breakpoint_Tables.pdf) and cefiderocol (FDC; MIC = 128 mg/L), along with ciprofloxacin, amikacin, and trimethoprim-sulfamethoxazole, while retaining susceptibility to eravacycline and tigecycline (Fig. 2A). Synergy testing results (by means of eTest/MIC strips, Fig. 2B) showed that initial MIC values of single agents dropped from 16 to 8 mg/L and from 8 to 3 mg/L for ATM and imipenem/relebactam (I/R), respectively. An ΣFICI value of 0.87 suggesting additivity was observed for the I/R+ATM combination, while meropenem/vaborbactam +ATM resulted in indifference (ΣFICI value of 1.75).

## CHALLENGING QUESTION

Which treatment strategy could be proposed on the basis of initial antimicrobial susceptibility patterns indicative of multiple resistance mechanisms?

- Colistin plus cefiderocol/fosfomycin
- Imipenem-relebactam-based plus a metallo-beta-lactamase (MBL) active betalactam (ATM)
- Tigecycline or eravacycline (+/- fosfomycin)
- Meropenem plus tigecycline

## TREATMENT AND OUTCOME

On day 5, the patient was not showing any significant improvement; fever persisted (always around 38°C), and inflammatory markers remained elevated. Following this poor clinical response, CZA was replaced by I/R 1.25 g q6h, while retaining ATM, in accordance with microbiology laboratory synergy testing results. On day 6 post-admission, standard dose tigecycline was added to the current combination regimen. The last febrile episode occurred on day 7, with a stable downward trend of inflammatory markers, blood cultures being persistently negative, and both drains being removed. Antibiotics were continued for 11 days without adverse events, and on day 18 post-admission, the patient was discharged home without clinical relapse throughout a 90-day follow-up. A rectal swab repeated after 5 months since hospital discharge did not reveal the presence of MDR pathogens.

Further testing and analyses were conducted to validate the clinical microbiology results and provide scientific rationale to support the choice of combining β-lactam agents (see Methods in Supplemental material).

Checkerboard broth dilution confirmed that I/R in combination with ATM had the lowest MIC and yielded the most favorable ΣFICI (0.09) (Table S1).

The strain was sent to a reference laboratory (University Hospital of Foggia, Italy) for whole-genome sequencing (WGS). Multilocus sequence typing (MLST) analysis indicated that this *E. coli* isolate as belonging to ST167, harboring *bla*_NDM-5_, *bla,*_TEM-1b_, and *bla*_CMY-148_ β-lactamases, *cirA* mutation resulting in a non-functional CirA protein, and a modified PBP3 with a YRIN insertion (Table S2 and Fig. S1).

**Fig 3:**
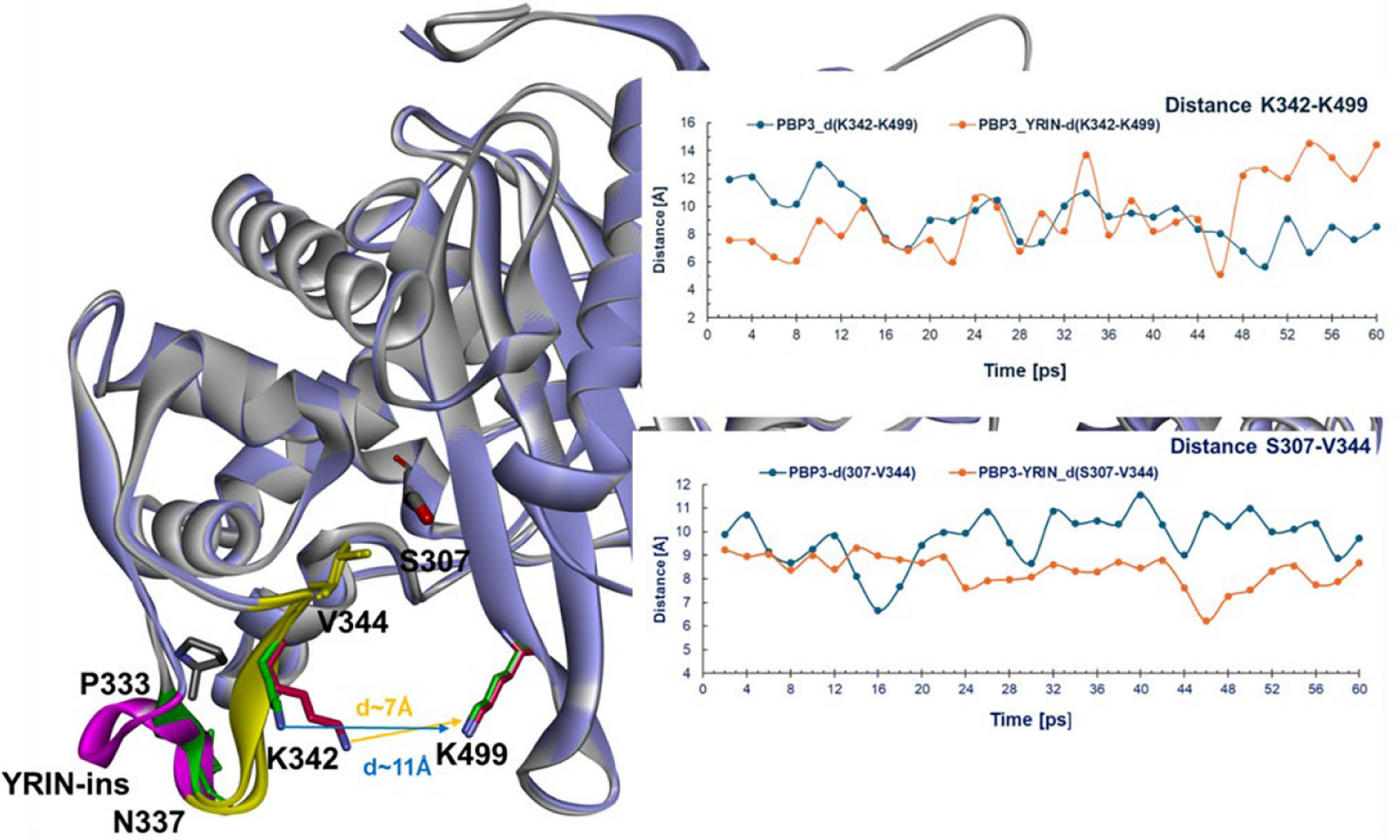
Superimposition of the initial structures of wild-type PBP3 (gray) and the PBP3-YRIN variant (light purple). The P333YRIN loop in wild-type PBP3 is shown in green, while the YRIN insertion in the variant is highlighted in magenta. The GHEIKDV loop (β-strand), part of the active site cavity, is colored yellow. In the top plots, the distance between K342 (green in PBP3, magenta in the variant) and K499 remains between 12–14 Å in the wild type and shortens to 6–8 Å in the variant. In the bottom plots, the initial distance between S307 and V344 increases from 9.5 Å to 10–11 Å in the wild-type, whereas in the variant, it decreases to below 9 Å and stabilizes at 7–8 Å over the course of the MDS.

To assess the impact of YRIN insertion in PBP3, a molecular dynamics simulation (MDS) was performed for wild-type PBP3 and the PBP3-YRIN variant, showing that the mutation significantly altered the shape and dynamics of the active site in the variant. Our MDS leads us to hypothesize that in wild-type PBP3, the K342–K499 distance remained 12–14 Å for the first 14 ps, while in the YRIN variant, it decreased to 6–8 Å, indicating active site constriction near the catalytic residue S307. After 14 ps, both stabilized at ~10 ± 1 Å (Fig. 3, top). Similarly, the S307–V344 distance in wild-type PBP3 increased from 9.5 Å to ~11 Å within 12 ps and stabilized, whereas in the variant, it decreased to ~7–8 Å, suggesting a more compact, less flexible active site (Fig. 3, bottom).

**Fig 4:**
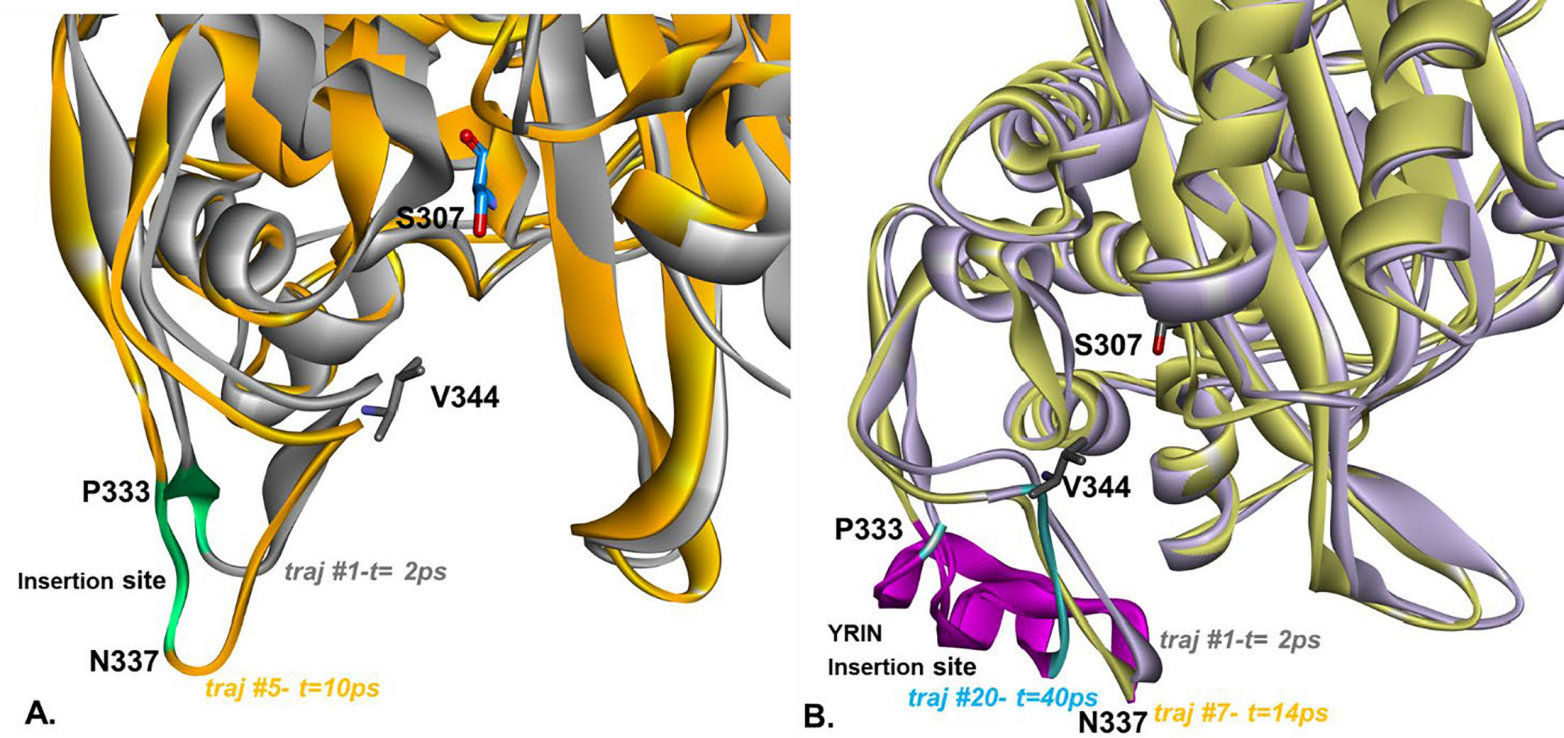
Representation of trajectory snapshots sampled during MDS at different time points, highlighting differences in flexibility and overall conformation between wild-type PBP3 (**A**) and the PBP3-YRIN variant (**B**). In PBP3, residue N337 shifts from its initial position (trajectory #1, 2 ps; colored gray) by up to 11 Å within the first 10 ps (trajectory #5; colored orange). In contrast, in the PBP3-YRIN variant, the same residue remains relatively stabilized, showing limited movement during the first 20 ps (trajectory #1, light purple; trajectory #7, yellow), before shifting up to 12 Å in the 10–20 ps interval (trajectory #20, light blue). The active site cavity of the PBP3-YRIN variant appears more constrained compared with the more flexible cavity in the wild type. The insertion site is colored green in wild-type PBP3 and magenta in the PBP3-YRIN variant.

MDS trajectories also suggested that distinct flexibility and conformational differences between PBP3 (Fig. 4A) and the PBP3-YRIN variant (Fig. 4B) were present. The variant’s higher global root mean square fluctuations, RMSF, (Fig. S2A) and lower local RMSF fluctuations (and RMS distances per residue), (Fig. S2B and S3) in the insertion loop indicate that the YRIN insertion stabilized the GHEIKDV β-strand, likely through new interactions (e.g., hydrogen bonds, hydrophobic packing) or steric constraints with the P333-YRIN loop. This stabilization constricts the active site, reducing its entrance from 11–12 Å to 6-8 Å (see Connolly surface, Fig. S4), limiting access for β-lactams with an R1 moiety like aztreonam and ceftazidime, but not imipenem.

Our case aligns with recent clinical reports with limited or no therapeutic options (2, 3), in which it becomes essential to reconsider “bench-to-bedside” testing strategies to assess the effectiveness of dual β-lactam combinations. Indeed, the most reliable treatment option consisted of combining a PBP2-binding carbapenem (I) and a β-lactamase inhibitor (R) with an antibiotic resistant to MBL inactivation (ATM). The therapy was initially proposed considering the AST profile showing ATM-AVI reduced susceptibility and high-level FDC resistance, suggestive of both enzymatic and PBP3 mutations, similar to that previously reported by Simner et al. (2). The aim of “target redundancy” was therefore carried out with this combination (6). Our analysis was later supported by checkerboard MIC testing, which revealed synergistic effects of all combinations. However, in our setting, the combination I/R +ATM was the one that allowed us to achieve the lowest MIC values with standard, non-toxic doses.

While still uncommon, PBP3 YRI-X amino acid sequence insertions are increasingly contributing to β-lactam resistance (7). These alterations are being implicated in conferring resistance to preclinical combinations prevalently targeting PBP3 in *Enterobacterales* (8), such as newly released β-lactams, including FDC. Conversely, carbapenem activity (especially I, preferentially binding to PBP2) is less impacted. Indeed, our MDS suggests that the restrained active site of the PBP3-YRIN variant likely prevents ATM, ceftazidime, and cefiderocol from forming a Michaelis-Menten complex by limiting/narrowing active site accessibility and flexibility. On the other hand, the smaller I can still access the constrained active site, maintaining its activity. Combination regimens conferring expanded PBP coverage have been proposed, also based on a “shielding” effect of double β-lactam therapy (9). Moreover, a recent report provides evidence supporting the combination of I/ATM in NDM-producing *Enterobacterales*, with an overall net effect of I based on its rapid penetration (10). Therefore, imipenem may reach its target PBPs before complete inactivation of the drug in the periplasmic space occurs, potentially contributing to early antibacterial effects. According to the “target redundancy” hypothesis, we also postulate that the potential interactions of REL with CMY (to protect ATM) and the nonproductive binding of ATM to NDM may allow just enough antibiotic to lower MICs. Hence, it is hence tempting to speculate that, in a similar fashion, PBP3 mutations may impact minimally on this combination.

How concerning or widespread is this? PBP3-mediated resistance has specifically been described in ST167 *E. coli* strains co-harboring an extensive β-lactamase arsenal, including NDM-5 and CMY-42 (11, 12), with the latter conferring resistance to CZA and FDC, possibly due to the similar structures of FDC and ceftazidime (13). In this case, the strain co-harbored a CMY-42 variant (CMY-148) with unknown hydrolysis profiles. Of note, CZA MICs by reference broth microdilution were extremely elevated, possibly due to extensive hydrolysis of the variant, coupled to other mechanisms, including reduced inhibition by AVI (14). Similar CMY variants with elevated resistance to CZA showed no impact on carbapenems (including imipenem [14]), as well as in other species, conferring cross-resistance to FDC (13).

Reports from China described 26 *E. coli* strains belonging to ST167, harboring NDM-5 variant MBL, along with PBP3 YRIN/YRIK insertions and displaying high-level resistance to FDC (15). A *cirA* premature stop codon occurred in 22/26 collected isolates, closely resembling our case. Similarly, the presented strain belonged to the ST167, suggestive of highly successful dissemination capacity (16, 17). However, to our knowledge, descriptions of such strains from low-risk, immunocompetent patients in non-endemic areas do not exist. The occurrence of similar encounters from the community is of remarkable concern and should call for raised awareness (1).

Limitations of this study include the lack of characterization of the CMY variant and its impact on β-lactam hydrolysis, as well as the potential impact of PBP protein expression in this isolate, which may impact therapy by binding and sequestration of β-lactams (18). While the impact of double β-lactam therapy is becoming more evident (19), the structural basis is still lacking, and the MDS provides limited sampling of these dynamics. Finally, other determinants, such as membrane permeability defects have not been ascertained.

In conclusion, the presented case highlights the importance of considering different β-lactam β-lactamase inhibitor combinations and the presence of other resistance determinants (e.g., PBP3 alterations) when managing extensive resistance patterns. Double β-lactam combination (including novel BLICs), upon reliable, real-time synergy testing, should be considered as an option, offering expanded PBP spectrum within PK/PD concentrations. To our knowledge so far, this is the first time a combination regimen based on I/R + ATM was used for the successful management of infections sustained by a pathogen displaying such resistance pattern. Further studies, also relying on sequencing and extensive biochemical evaluation, are ongoing for a deeper understanding and efficient management of similar cases.

## Data Availability

Sequencing reads and *de novo* whole-genome assembly were deposited to NCBI under BioProject PRJNA1088412.

## SUPPLEMENTAL MATERIAL

Supplemental material aac.00887-25-s0001.docx Tables S1 and S2; Fig. S1 to S4; Supplemental methods.

ASM does not own the copyrights to Supplemental Material that may be linked to, or accessed through, an article. The authors have granted ASM a non-exclusive, world-wide license to publish the Supplemental Material files. Please contact the corresponding author directly for reuse.

## References

[1] Linkevicius M, Bonnin RA, Alm E, Svartström O, Apfalter P, Hartl R, Hasman H, Roer L, Räisänen K, Dortet L, et al.. 2023. Rapid cross-border emergence of NDM-5-producing Escherichia coli in the European Union/European economic area, 2012 to June 2022. Euro Surveill 28. doi:10.2807/1560-7917.ES.2023.28.19.2300209 PMC10176832 37166762

[2] Simner PJ, Bergman Y, Conzemius R, Jacobs E, Tekle T, Beisken S, Tamma PD. 2023. An NDM-producing Escherichia coli clinical isolate exhibiting resistance to cefiderocol and the combination of ceftazidime-avibactam and aztreonam: another step toward pan-β-lactam resistance. Open Forum Infect Dis 10:ofad276. doi:10.1093/ofid/ofad276 37416757 PMC10319620

[3] Senchyna F, Murugesan K, Rotunno W, Nadimpalli SS, Deresinski S, Banaei N. 2024. Sequential treatment failure with aztreonam-ceftazidime-avibactam followed by cefiderocol due to preexisting and acquired mechanisms in a New Delhi metallo-β-lactamase-producing Escherichia coli causing fatal bloodstream infection. Clin Infect Dis 78:1425–1428. doi:10.1093/cid/ciad759 38289725

[4] Le Terrier C, Nordmann P, Buchs C, Poirel L. 2024. Effect of modification of penicillin-binding protein 3 on susceptibility to ceftazidime-avibactam, imipenem-relebactam, meropenem-vaborbactam, aztreonam-avibactam, cefepime-taniborbactam, and cefiderocol of Escherichia coli strains producing broad-spectrum β-lactamases. Antimicrob Agents Chemother 68:e0154823. doi:10.1128/aac.01548-23 38415988 PMC10989025

[5] Sader HS, Carvalhaes CG, Kimbrough JH, Mendes RE, Castanheira M. 2024. Activity of aztreonam-avibactam against Enterobacterales resistant to recently approved beta-lactamase inhibitor combinations collected in Europe, Latin America, and the Asia-Pacific Region (2020-2022). Int J Antimicrob Agents 63:107113. doi:10.1016/j.ijantimicag.2024.107113 38354826

[6] Dousa KM, Kurz SG, Taracila MA, Bonfield T, Bethel CR, Barnes MD, Selvaraju S, Abdelhamed AM, Kreiswirth BN, Boom WH, Kasperbauer SH, Daley CL, Bonomo RA. 2020. Insights into the l,d-transpeptidases and d,d-carboxypeptidase of Mycobacterium abscessus: ceftaroline, imipenem, and novel diazabicyclooctane inhibitors. Antimicrob Agents Chemother 64:e00098-20. doi:10.1128/AAC.00098-20 32393499 PMC7526840

[7] Sethuvel DPM, Bakthavatchalam YD, Karthik M, Irulappan M, Shrivastava R, Periasamy H, Veeraraghavan B. 2023. β-lactam resistance in ESKAPE pathogens mediated through modifications in penicillin-binding proteins: an overview. Infect Dis Ther 12:829–841. doi:10.1007/s40121-023-00771-8 36877435 PMC10017896

[8] Liu X, Li Z, Zhang F, Yang X, Lei Z, Li C, Wu Y, Zhao J, Zhang Y, Hu Y, Shen F, Wang P, Yang J, Liu Y, Shi H, Lu B. 2025. In vitro antimicrobial activity of six novel β-lactam and β-lactamase inhibitor combinations and cefiderocol against NDM-producing Enterobacterales in China. Int J Antimicrob Agents 65:107407. doi:10.1016/j.ijantimicag.2024.107407 39672348

[9] Siriyong T, Murray RM, Bidgood LE, Young SA, Wright F, Parcell BJ, Voravuthikunchai SP, Coote PJ. 2019. Dual β-lactam combination therapy for multi-drug resistant Pseudomonas aeruginosa infection: enhanced efficacy in vivo and comparison with monotherapies of penicillin-binding protein inhibition. Sci Rep 9:9098. doi:10.1038/s41598-019-45550-z 31235728 PMC6591303

[10] Kaur JN, Singh N, Smith NM, Klem JF, Cha R, Lang Y, Chen L, Kreiswirth B, Holden PN, Bulitta JB, Tsuji BT. 2024. Next generation antibiotic combinations to combat pan-drug resistant Klebsiella pneumoniae. Sci Rep 14:3148. doi:10.1038/s41598-024-53130-z 38326428 PMC10850076

[11] Sato T, Ito A, Ishioka Y, Matsumoto S, Rokushima M, Kazmierczak KM, Hackel M, Sahm DF, Yamano Y. 2020. Escherichia coli strains possessing a four amino acid YRIN insertion in PBP3 identified as part of the SIDERO-WT-2014 surveillance study. JAC Antimicrob Resist 2:dlaa081. doi:10.1093/jacamr/dlaa081 34223033 PMC8210206

[12] Sadek M, Juhas M, Poirel L, Nordmann P. 2020. Genetic features leading to reduced susceptibility to aztreonam-avibactam among metallo-β-lactamase-producing Escherichia coli isolates. Antimicrob Agents Chemother 64:e01659–20. doi:10.1128/AAC.01659-20 32988825 PMC7674043

[13] Kawai A, Shropshire WC, Suzuki M, Borjan J, Aitken SL, Bachman WC, McElheny CL, Bhatti MM, Shields RK, Shelburne SA, Doi Y. 2024. Structural insights into the molecular mechanism of high-level ceftazidime–avibactam resistance conferred by CMY-185. mBio 15. doi:10.1128/mbio.02874-23 PMC10865806 38179965

[14] Xu T, Wu W, Huang L, Liu B, Zhang Q, Song J, Liu J, Li B, Li Z, Zhou K. 2024. Novel plasmid-mediated CMY variant (CMY-192) conferring ceftazidime-avibactam resistance in multidrug-resistant Escherichia coli. Antimicrob Agents Chemother 68:e0090624. doi:10.1128/aac.00906-24 39470201 PMC11619348

[15] Wang Q, Jin L, Sun S, Yin Y, Wang R, Chen F, Wang X, Zhang Y, Hou J, Zhang Y, Zhang Z, Luo L, Guo Z, Li Z, Lin X, Bi L, Wang H. 2022. Occurrence of high levels of cefiderocol resistance in carbapenem-resistant Escherichia coli before its approval in China: a report from China CRE-network. Microbiol Spectr 10:e02670–21. doi:10.1128/spectrum.02670-21 35481835 PMC9241927

[16] Amadesi S, Gatti M, Rinaldi M, Pea F, Viale P, Gaibani P. 2024. Novel CMY-186 variant conferring cross-resistance to cefiderocol and ceftazidime/avibactam in Klebsiella pneumoniae from a critically ill patient during cefiderocol and ceftazidime/avibactam treatment. Int J Antimicrob Agents 63:107107. doi:10.1016/j.ijantimicag.2024.107107 38325723

[17] Giufrè M, Errico G, Accogli M, Monaco M, Villa L, Distasi MA, Del Gaudio T, Pantosti A, Carattoli A, Cerquetti M. 2018. Emergence of NDM-5-producing Escherichia coli sequence type 167 clone in Italy. Int J Antimicrob Agents 52:76–81. doi:10.1016/j.ijantimicag.2018.02.020 29501819

[18] Montaner M, Lopez-Argüello S, Oliver A, Moya B. 2023. PBP target profiling by β-lactam and β-lactamase inhibitors in intact Pseudomonas aeruginosa: effects of the intrinsic and acquired resistance determinants on the periplasmic drug availability. Microbiol Spectr 11:e0303822. doi:10.1128/spectrum.03038-22 36475840 PMC9927461

[19] Tao L, Dahlquist A, Harris H, Jacobs E, Wenzler E, Simner PJ, Humphries R. 2024. Multicenter evaluation of activity of aztreonam in combination with avibactam, relebactam, and vaborbactam against metallo-β-lactamase-producing carbapenem-resistant gram-negative bacilli. Antimicrob Agents Chemother 68:e0069324. doi:10.1128/aac.00693-24 39158279 PMC11459955

