# Supplementary material for: Optimizing target inactivation to treat multidrug-resistant Escherichia coli with NDM and PBP3 mutations: “going the extra mile”
Source: Antimicrob Agents Chemother. 2026 Feb 20;70(5):e00887-25. doi: 10.1128/aac.00887-25 (PMC13148025; doi:10.1128/aac.00887-25)
Supplement: Supplemental material — Tables S1 and S2; Fig. S1 to S4; Supplemental methods. [file aac.00887-25-s0001.docx]

**Material and methods**

**Antimicrobial susceptibility testing (AST)**

Initial susceptibility testing was performed by routine diagnostics methods. Briefly, samples were processed according to the manufacturer’s instructions by means of automated systems (Vitek 2, BioMérieux France). All off-panel testing were also carried out in accordance with manufacturing instructions (Liofilchem Roseto Degli Abruzzi, BioMérieux France). Results were interpreted according to EUCAST rules and breakpoints (<https://www.eucast.org/fileadmin/src/media/PDFs/EUCAST_files/Breakpoint_tables/v_15.0_Breakpoint_Tables.pdf>).

Synergy testing for the combination imipenem-relebactam/aztreonam (I/R+ATM) was performed by means of eTest MIC strips (performed in triplicate), as previously described [1], after calculating the single drugs MICs by means of both broth dilution and eTest, within an overall 72-hour time. To assess synergy/antagonism, the FICI index was calculated as described in the literature [2] for both I/R+ATM and meropenem-vaborbactam/aztreonam (M/V+ATM) combinations (other combinations were not locally available).

**Sequencing and Characterization**

The strain was sent to a reference laboratory (University Hospital of Foggia, Italy) for further testing and genomic characterization by whole-genome sequencing (WGS). Previous AST results were confirmed by reference broth microdilution (BMD) method (CLSI, M07Ed11, 2018), which were repeated in triplicate in order to understand discrepancies with the results obtained in the local laboratory (Fig. 2a). WGS was run using the MinION platform (Oxford Nanopore Technologies, Oxford, UK). Genome assembly was performed using the Flye software version 2.8.3. Molecular typing and resistance gene content were investigated using the PubMLSTdatabase (available at [https://pubmlst.org](about:blank)) and ABRicate software version 1.0.1 (available at [https://github.com/tseemann/ABRICATE](about:blank)), respectively.

**Checkerboard Testing** Checkerboard broth dilution method for assessing synergy was performed according to reference methods and as previously described [3]. Briefly, each antibiotic in the combination was diluted in a 96 well plate from wells 1-12 or wells A-G separately. To summarize, IMI was diluted across the plate in columns 1 – 12 from 1024 mg/L – 0.5 mg/L, while the ATM were diluted down the plate in rows A – G from 512 mg/L – 4 mg/L in a final volume of 100ul. Finally, 100 ul of cells with inhibitors are added to each well from a diluted 0.5 MacFarland standard to be approximately 5 x 10^5CFU/ml. The final concentration of ATM is 256-2 mg/L and of IMI 256-0.125 mg/L and 4mg/L of relebactam in all wells. MIC controls were done at the same time with the same final concentration and inoculum (**Table S1A-B**)

**Molecular Dynamic Simulation and analysis -**

The crystal structure of PBP3 from *E. coli* (PDB: 7ONO) was used for molecular modeling and simulation. A molecular model of PBP3 with a YRIN insertion after residue Pro333 was generated using SWISS-MODEL homology-modeling server (https://swissmodel.expasy.org/). Both the wild-type and PBP3-YRIN models were prepared and analyzed using Discovery Studio Client 2022 (Dassault Systèmes BIOVIA, San Diego: Dassault Systèmes, 2016). The structures were energy-minimized using a Conjugate Gradient method, with CHARMM force field, to an RMS gradient of 0.002 kcal/(mol x Å). Generalized Born with a simple Switching (GBSW) solvation model was employed, and long-range electrostatics were treated using a Particle Mesh Ewald (PME) method with periodic boundary condition. The SHAKE algorithm was applied to constrain bonds involving hydrogen atoms, enabling a 2 fs time step. To equilibrate the structures, a 60 ps molecular dynamic simulation (MDS) was performed at 300 K. Following the equilibration, the trajectories were processed and analyzed using Discovery Studio protocols. Distances between active site residues (K342-K499 and S307-V344) were monitored during the MDS to assess local movement and changes. To evaluate structural changes induced by the YRIN insertion, the root mean square deviation (RMSD) was calculated for all conformations relative to the initial structure after trajectory superposition, and RMSD values were reported for each residue. To investigate local dynamics of the insertion loop and active site residues, the root mean square fluctuation (RMSF) was calculated relative to the average structure for each residue of both PBP3 and the variant.

**Molecular Dynamics Simulation: Discussion and Results**

Higher global RMSF in the PBP3-YRIN variant (**Figure S2A**) indicates tertiary structure disruption, increasing conformational variability outside the active site and PYRIN-YRIN loop (P333–N337 plus YRIN insertion). However, RMS fluctuations in the insertion loop and GHEIKDV β-strand residues, including K342, are ~1 Å lower in the variant than in the wild-type PYRIN loop (P333–N337) and GHEIKDV β-strand (**Figure S2B**), suggesting reduced local flexibility. This stabilization, likely due to new interactions (e.g., hydrogen bonds, hydrophobic packing) or steric constraints with the insertion loop, limits active site residue mobility. Similarly, RMSD in these regions decreases from 10–11 Å in wild-type to 7–8 Å in the variant (**Figure S3**), indicating stabilized loop and β-strand conformations, though residue N337 in the variant’s insertion loop shows increased RMSD (7–12 Å in 40–60 ps), suggesting localized flexibility. This stabilization constricts the active site, as shown by the Connolly surface representation (**Figure S4**).

**References**

1. Rawson TM, Brzeska-Trafny I, Maxfield R et al. A practical laboratory method to determine ceftazidime-avibactam-aztreonam synergy in patients with New Delhi metallo-beta-lactamase (NDM)–producing Enterobacterales infection. J Glob Antimicrob Resist, Volume 29, 2022, Pages 558-562, ISSN 2213-7165.
2. Hall MJ, Middleton RF, Westmacott D. The fractional inhibitory concentration (FIC) index as a measure of synergy. J Antimicrob Chemother. 1983 May;11(5):427-33.
3. Hsieh MH, Yu CM, Yu VL, Chow JW. Synergy assessed by checkerboard. A critical analysis. Diagn Microbiol Infect Dis 1993; 16:343–9.

**Table S1**

**A**: Checkerboard testing results

| **Antibiotic** | **MICa (mg/L)** | **BL-BLIC MIC + AZT combination (mg/L)** | **ATM MIC + BL-BLIC combination (mg/L)** | Σ**FICI** | **EUCAST breakpoint (mg/L)** |
| --- | --- | --- | --- | --- | --- |
| ATM | 128 | - | - | - | 8 |
| MVB | 32 | 4 | 16 | 0.25 | 8 |
| I/R | 16 | 1 | 4 | 0.09 | 2 |
| CZA | 8192 | 16 | 8 | 0.15 | 8 |

MICa= MIC values of single agent

BLIC= Beta Lactam Beta Lactamase Inhibitor Combination

ΣFICI= fractional inhibitory concentration index calculated according to Hall et al (6)

**B**: Schematic of checkerboard 96 well plate showing the final concentration of two antibiotics in each well after inoculation

Drug A is shown in blue; drug B is shown in red. Column 12 represents the no-drug control


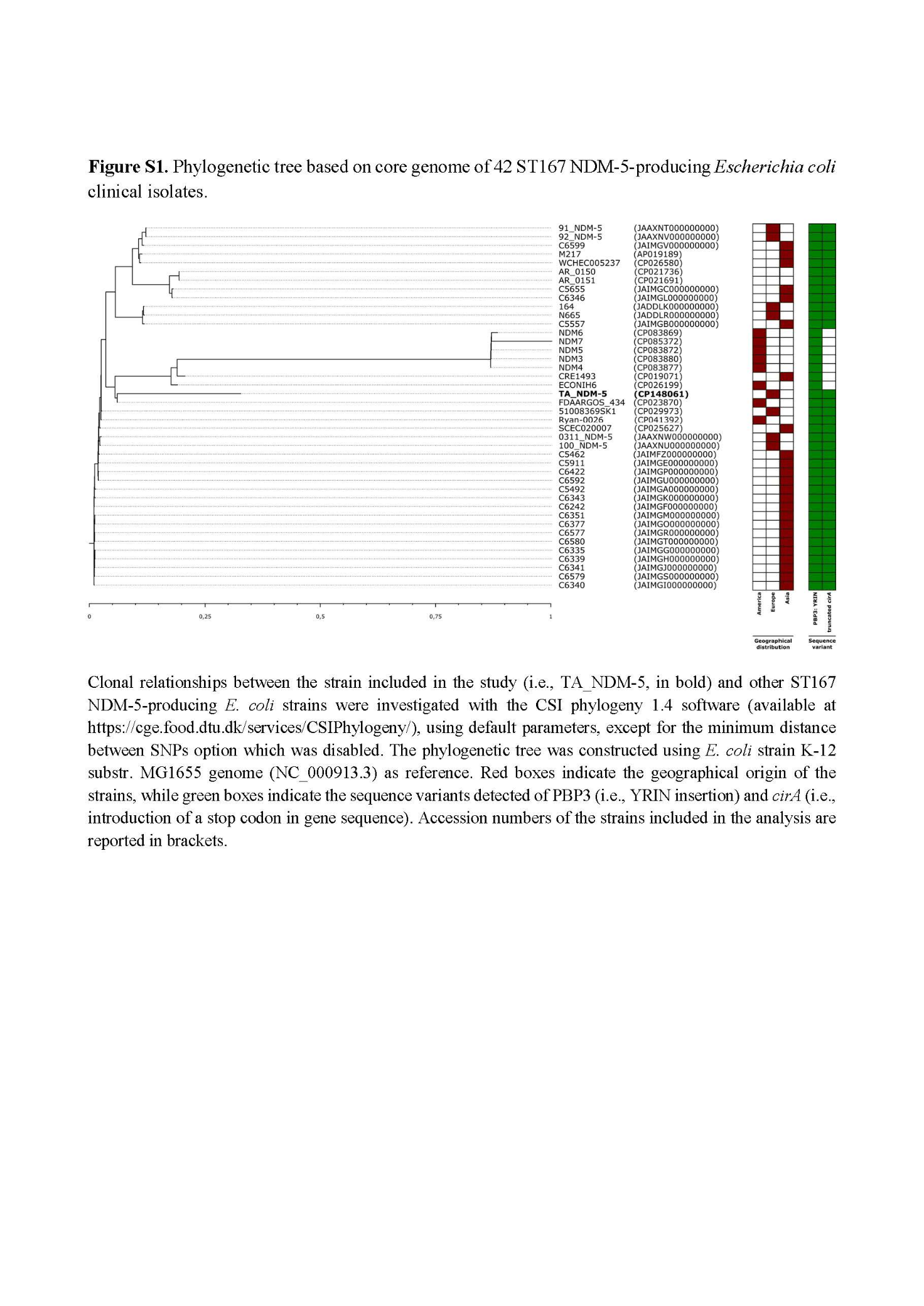


**Table S2.** Antimicrobial resistance genes of the ST167 NDM-5-producing *Escherichia coli* strain detected by resistome analysis.

| **Antibiotic class** | **Resistance genes** | **Genome localization^a^** | **Coverage (%)** | **Identity (%)** | **Accession no.** |  |
| --- | --- | --- | --- | --- | --- | --- |
|  |  |  |  |  |  |  |
| Aminoglycosides |  |  |  |  |  |  |
|  | *aac3-Iid* | IncI1-α [ST26] | 99.88 | 99.88 | NG_047251.1 |  |
|  | *aadA1* | IncI1-α [ST26] | 100 | 100 | NG_052146.1 |  |
|  | *aadA2* | IncFII [F2:A-:B-] | 98.48 | 100.00 | NG_047343.1 |  |
|  | *rmtB* | IncFII [F2:A-:B-] | 99.74 | 99.73 | NG_048058.1 |  |
| β-lactams |  |  |  |  |  |  |
|  | *bla*_EC-15_ | Chr | 99.91 | 98.50 | NG_049081.1 |  |
|  | *bla*_CMY-148_ | IncI-γ | 99.91 | 99.91 | NG_054681.1 |  |
|  | *bla*_TEM-1_ | IncI1-α [ST26], IncFII [F2:A-:B-] | 99.88, 100 | 99.88, 100 | NG_050145.1 |  |
|  | *bla*_NDM-5_ | IncFII [F2:A-:B-] | 100 | 100 | NG_049337.1 |  |
| Macrolides |  |  |  |  |  |  |
|  | *mph(A)* | IncFII [F2:A-:B-] | 100 | 98.60 | NG_047986.1 |  |
|  | *erm(B)* | IncFII [F2:A-:B-] | 100 | 99.86 | NG_047804.1 |  |
| Tetracyclines |  |  |  |  |  |  |
|  | *tet(M)* | IncI1-α [ST26] | 99.90 | 99.11 | NG_048244.1 |  |
|  | *tet(A)* | IncI1-α [ST26] | 99.75 | 99.75 | NG_048154.1 |  |
| Sulfonamides |  |  |  |  |  |  |
|  | *sul1* | Chr | 100.00 | 100.00 | NG_048082.1 |  |
| Trimethoprim |  |  |  |  |  |  |
|  | *dfrA12* | IncFII [F2:A-:B-] | 100.00 | 100.00 | NG_047689.1 |  |
|  |  |  |  |  |  |  |


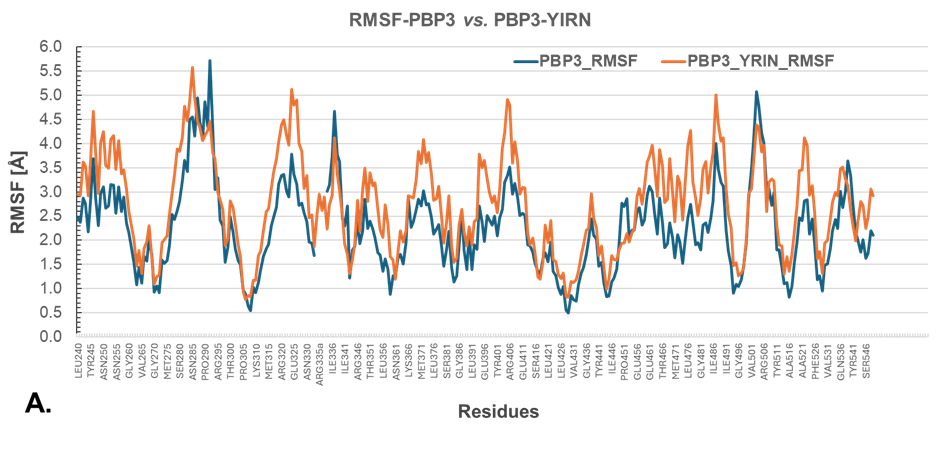


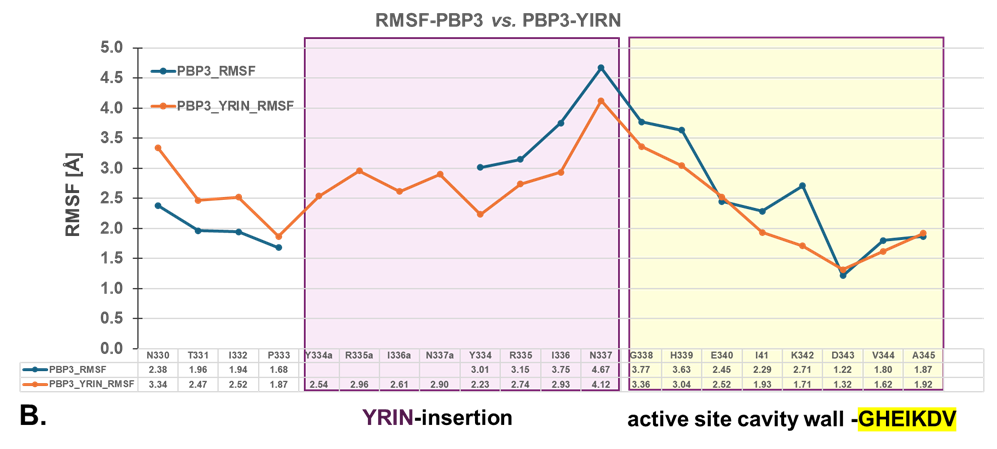


**Figure S2**: Superimposition of per-residue root mean square fluctuation (RMSF) values for wild-type PBP3 (blue) and the PBP3-YRIN variant (orange) over the 60 ps MDS reveals greater overall flexibility in the variant (**A**), likely due to the YRIN insertion. However, in the PYRIN-YRIN loop and GHEIKDV β-strand region (**B**), the variant shows RMS fluctuations that are 0.5–1 Å lower, indicating reduced mobility in this region. These findings suggest that this localized stabilization may ontribute to increased rigidity of the PBP3-YRIN variant's active site.

**Figure S3**: RMSD (root mean square deviation per residue)
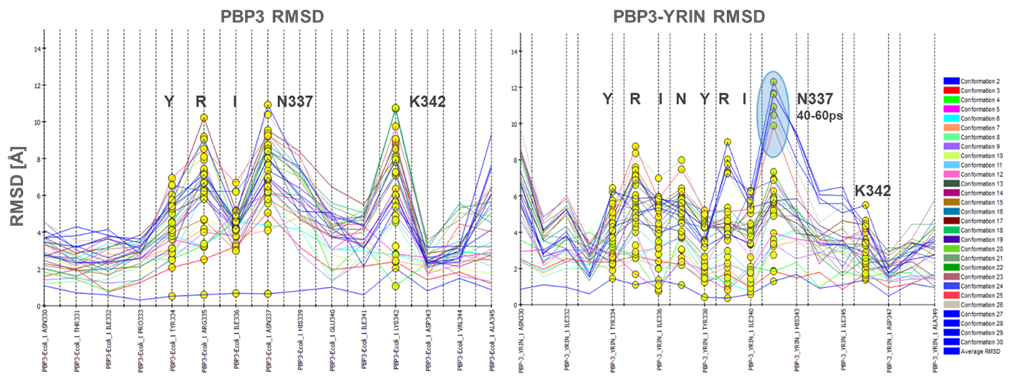


RMSD (root mean square deviation per residue) of the PYRIN loop (P333–N337) and GHEIKDV β-strand (including K342) in wild-type PBP3, and the PYRIN-YRIN loop (P333–N337 plus YRIN insertion) and GHEIKDV β-strand in the PBP3-YRIN variant during a 60 ps MDS) Trajectories were sampled every 2 ps and compared to the initial structure. The PYRIN-YRIN loop and GHEIKDV β-strand in the variant exhibit lower RMSD values (7–8 Å) than the corresponding regions in wild-type PBP3 (10–11 Å), indicating increased stability consistent with active site constriction. Notably, residue N337 in the variant shows increased RMSD in the final 20 ps (increasing from ~7 Å to ~12 Å), suggesting localized flexibility at the end of the loop.


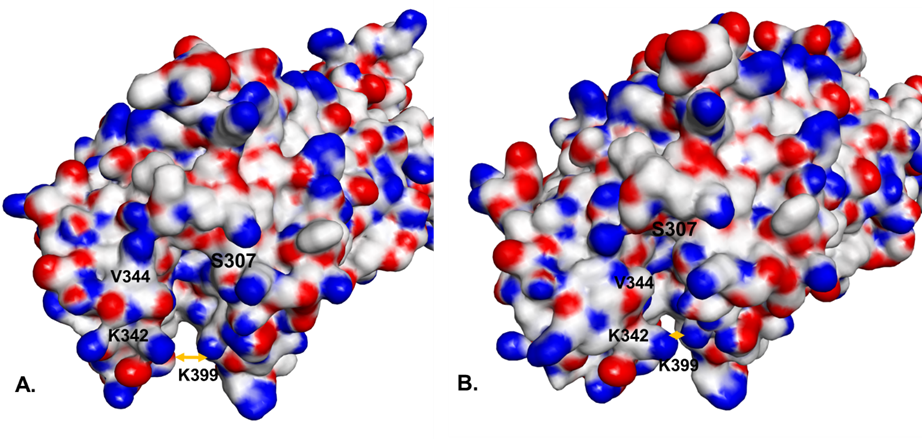


**Figure S4:** Connolly surface representation of PBP3 (**A**) and the PBP3-YRIN variant (B). In the variant, the active site is constricted due to movement of K342 (part of the GHEIKDV β-strand lining the active site cavity) toward K499. As a result, the entrance to the active site narrows from 11–12 Å in wild-type PBP3 to 6–8 Å in the YRIN variant.
